# COVID, complement, and the brain

**DOI:** 10.3389/fimmu.2023.1216457

**Published:** 2023-07-18

**Authors:** Sonia I. Vlaicu, Alexandru Tatomir, Jacob Cuevas, Violeta Rus, Horea Rus

**Affiliations:** ^1^Department of Neurology, University of Maryland, School of Medicine, Baltimore, MD, United States; ^2^Department of Medicine, Medical Clinic Nr. 1, “Iuliu Hatieganu” University of Medicine and Pharmacy, Cluj-Napoca, Romania; ^3^Department of Medicine, Division of Rheumatology and Clinical Immunology, University of Maryland, School of Medicine, Baltimore, MD, United States; ^4^Neurology Service, Baltimore Veterans Administration Medical Center, Baltimore, MD, United States

**Keywords:** COVID, brain, complement system, C5b-9, endothelial cells, cognitive dysfunction

## Abstract

The brains of COVID-19 patients are affected by the SARS-CoV-2 virus, and these effects may contribute to several COVID-19 sequelae, including cognitive dysfunction (termed “long COVID” by some researchers). Recent advances concerning the role of neuroinflammation and the consequences for brain function are reviewed in this manuscript. Studies have shown that respiratory SARS-CoV-2 infection in mice and humans is associated with selective microglial reactivity in the white matter, persistently impaired hippocampal neurogenesis, a decrease in the number of oligodendrocytes, and myelin loss. Brain MRI studies have revealed a greater reduction in grey matter thickness in the orbitofrontal cortex and parahippocampal gyrus, associated with a greater reduction in global brain size, in those with SARS-CoV-2 and a greater cognitive decline. COVID-19 can directly infect endothelial cells of the brain, potentially promoting clot formation and stroke; complement C3 seems to play a major role in this process. As compared to controls, the brain tissue of patients who died from COVID-19 have shown a significant increase in the extravasation of fibrinogen, indicating leakage in the blood–brain barrier; furthermore, recent studies have documented the presence of IgG, IgM, C1q, C4d, and C5b-9 deposits in the brain tissue of COVID-19 patients. These data suggest an activation of the classical complement pathway and an immune-mediated injury to the endothelial cells. These findings implicate both the classical and alternative complement pathways, and they indicate that C3b and the C5b-9 terminal complement complex (membrane attack complex, MAC) are acting in concert with neuroinflammatory and immune factors to contribute to the neurological sequelae seen in patients with COVID.

## Introduction

SARS-CoV-2 infection affects not only the lungs but also the brain. Many studies have shown that the brains of COVID-19 patients are affected by the virus and that these effects may have contributed to the sequelae they experienced, including cognitive dysfunction (1–3). Most people recover within weeks from infection with SARS-CoV-2, the virus that causes COVID-19, but others may experience new or lingering symptoms even after recuperating from COVID-19. These lingering symptoms after COVID-19 are labeled by the Centers for Disease Control (CDC) as “long COVID”. People who develop long COVID usually had a more severe form of COVID-19 including hospitalization, had underlying health conditions, and/or experienced multisystem inflammatory syndrome during COVID.

Long COVID neurological sequels include ischemic and hemorrhagic stroke, cognition and memory disorders, peripheral nervous system disorders, migraine, seizures, extrapyramidal and movement disorders, mental health disorders, musculoskeletal disorders, sensory disorders, Guillain–Barré syndrome, and encephalitis or encephalopathy (4). One of the most compelling studies was recently published by Xu et al., in which the national healthcare databases of the US Department of Veterans Affairs were used to build a cohort of 154,068 individuals with COVID-19; 5,638,795 contemporary controls and 5,859,621 historical controls and estimate, in these groups, the risks and burdens of incident neurologic disorders at 12 months following acute SARS-CoV-2 infection (4). Xu et al. have shown that in the post-acute phase of COVID-19, there is an increased risk for neurologic sequelae. Authors of this study estimated that the hazard ratio for any neurologic sequela was 1.42 (95% confidence intervals, 1.38 and 1.47) and the burden 70.69 (95% confidence intervals, 63.54 and 78.01) per 1,000 persons at 12 months (4). The risks and burdens were elevated even in those who did not require hospitalization during acute COVID-19. This study, however, was limited in the fact that the cohort included only white males. Nevertheless, these results showed the long-term effects of COVID-19 on the brain and provide a more comprehensive assessment of the devastating long-term neurologic consequences of COVID-19 (4).

Recently Schulte et al. (5) have established an open global collaboration to elucidate genetic risk factors for long COVID called The Long COVID Host Genetics Initiative (46 studies across 23 countries). The first analysis involved genome-wide association analyses (GWAS) to compare long COVID patients (N=1,588) to individuals who recovered from COVID within 3 months (N=96,714), as well as to population controls (N=800,478). GWAS of individual cohorts suggested potential variants associated with long COVID but did not achieve genome-wide statistical significance. In one subgroup, in samples from Finland with 2,018 participants, 50 (2.4%) patients were found to have a long COVID diagnosis. In this subgroup, the authors found that long COVID was associated with previous autoimmune diseases (p = 0.026). Although no locus of genome-wide significance has been identified to date, this initiative will make it possible (by increasing the sample size) to identify new genetic risk factors for long COVID in the future (5). Many genetic susceptibility studies in COVID-19 have focused on the role of genetic factors in severe COVID. These studies are beyond the scope of this review and were reviewed recently by Cappadonna et al. (6).

In this review we present the most recent advances regarding the neurobiology of COVID-19 and the role of immune and glial interactions, as well as the role of the complement system in long COVID.

### Neurobiology of COVID

As mentioned above, COVID survivors frequently experience persistence of neurological symptoms, including fatigue and cognitive impairment, which are among the most-reported long COVID symptoms. Most people recover within weeks from infection with SARS-CoV-2, the virus that causes COVID-19, but others may experience new or lingering symptoms even after recuperating from COVID-19. These lingering symptoms after COVID-19 are labeled by the CDC as “long COVID,” to which white matter microglial reactivity and consequent neural dysregulation are central.

The neurobiological effects have been studied in both animal models and in humans. The most important studies are summarized below. Fernández-Castañeda et al. have recently investigated the neurobiological effects of respiratory SARS-CoV-2 infection in mice and humans and found white matter-selective microglial reactivity (7). Following mild respiratory COVID in mice, they noted a persistently impaired hippocampal neurogenesis, fewer oligodendrocytes, and myelin loss. When examining oligodendroglial lineage cells in the subcortical white matter, these authors found that by 7 weeks after infection, a mild decrease (∼10%) in the number of oligodendrocyte precursor cells was evident and was associated with a loss of approximately one-third of the total number of oligodendrocytes by 7 days post-infection. This depletion in mature oligodendrocytes persisted until at least 7 weeks post-infection. They also found elevated cerebrospinal fluid (CSF) cytokine/chemokine levels, including those of C-C motif chemokine 11 (CCL11). Systemic CCL11 administration specifically caused hippocampal microglial reactivity and impaired neurogenesis.

To further explore the relevance of elevated CCL11 levels to the cognitive sequelae of COVID-19 infection in humans, these same authors examined CCL11 cytokine levels in the plasma of people suffering from long COVID, with and without cognitive symptoms. The patients included in the study experienced a relatively mild course of SARS-CoV-2 infection, without hospitalization in more than 90% of subjects. They documented elevated CCL11 levels in the plasma of those with long COVID who exhibited cognitive deficits (7). CCL11 increases with aging and was found to be associated with cognitive dysfunction. As compared to SARS-CoV-2, mild respiratory influenza in mice caused similar patterns of white-matter-selective microglial reactivity, oligodendrocyte loss, impaired neurogenesis, and elevated CCL11 at early time points, but after influenza, only elevated CCL11 and hippocampal pathology persisted. Taken together, these findings demonstrate that even mild respiratory infection with SARS-CoV-2 can result in persistent neuroinflammatory changes and cognitive changes (7). All these studies were done using the earlier strain of the SARS-CoV2 virus, and the relevance of their findings to newer stains of the virus are unknown. Also, the findings need to be replicated in studies using a larger number of patients.

The role of spike protein in cognitive defunction after COVID-19 was recently investigated by Fontes-Dantas et al. (8). When mice were infused with spike protein late but not early microgliosis was induced by spike protein in the dentate gyrus hippocampal subregion. In addition, SARS-CoV-2 Spike protein induces C1q mediated synaptic phagocytosis by microglia. C1q blockage prevented the decrease in synapses in hippocampus in spike infused mice. These data suggest that cognitive dysfunction is related to the C1q mediated synaptic phagocytosis by microglia. Toll-like receptor 4 (TLR4) activation also plays a significant role in mediation of cognitive dysfunction and synaptic pruning. The authors examined two different SNPs (rs10759931 and rs2737190) in 86 human subjects with mild COVID; half of these patients presented with significant post-COVID-19 cognitive dysfunction. The study found that individuals carrying the *TLR4*-2604G>A (rs10759931) GG homozygous genotype demonstrated a significantly higher risk for developing cognitive dysfunction following SARS-CoV-2 infection. This study supports the role for TLR4 genetic status and SARS-CoV-2 cognitive outcomes in patients with long COVID.

Cognitive decline in humans has been investigated using EEG, brain MRIs and PET scans. In a recent cognition study of EEG and brain MRI scans in patients with COVID at baseline (within 2 months from COVID-19 diagnosis), 53% and 28% of patients showed cognitive and psychopathological disturbances, respectively, along with executive dysfunction. During the acute phase of COVID-19, 85.7% of patients were treated in the hospital. As compared to healthy controls, there was a correlation between executive performance and both higher regional current density and connectivity at the delta band on EEG in patients with COVID-19; on brain MRI, they also showed a greater white matter hyperintensity that correlated with verbal memory deficits. A reduction in cognitive impairment and delta band EEG connectivity was observed over time, with psychopathological symptoms persisting. Patients with acute dysgeusia/hyposmia showed a lesser improvement in memory tests than did those without these conditions. Furthermore, a lower EEG delta band at baseline predicted worse cognitive functioning at follow-up (9).

To gain more insight on the SARS-CoV-2 effect on the brain, Douaud and coworkers investigated brain changes in 785 participants who were imaged twice using MRI, including 401 participants who tested positive for infection with SARS-CoV-2 between their two scans. The authors found a greater reduction in grey matter thickness and tissue contrast in the orbitofrontal cortex and parahippocampal gyrus, associated with a greater reduction in global brain size, in the SARS-CoV-2-positive individuals as well as a greater cognitive decline. In addition, they reported greater changes in markers of tissue damage in regions that are functionally connected to the primary olfactory cortex. These imaging and cognitive effects were still observed after the 15 patients who had been hospitalized were excluded. One of the limitations of this study was the lack of stratification of the severity of the cases. Longer-term studies are clearly needed to investigate whether these effects persist in the long term (10).

In a recent 18-fluorodeoxyglucose positron emission tomography (^18^FDG-PET) study, patients with long COVID exhibited hypometabolism in several brain regions (the bilateral rectal/orbital gyrus, including the olfactory gyrus; the right temporal lobe, including the amygdala and the hippocampus; the bilateral pons/medulla brainstem and cerebellum) and was associated with persistent symptoms, such as hyposmia/anosmia and cognitive impairment (11). Patients included in this study had more the 3 weeks since COVID-19 diagnosis and complained of fatigue and symptoms of possible neurological origin. Patients with lesions on CT or MRI of the brain were excluded from the study (11).

Arterial spin labeling (ASL) is a relatively new MRI technique used to measure cerebral blood flow, which is increasingly being used to assess brain perfusion in subjects affected by neurological diseases. Post COVID-19 brain perfusion alterations were investigated by means of ASL in 24 patients with persistent cognitive complaints in the post COVID-19 period. Patients with moderate-to-severe COVID-19 disease, defined as patients with clinical and radiologic evidence of lower respiratory tract infection or hospitalized for COVID-19 were excluded from the study. The findings identified a significant cerebral hypoperfusion pattern in the post-COVID-19 group, predominantly affecting the frontal cortex, as well as the parietal and temporal cortex. Interestingly, the hypoperfusion areas documented by the authors in the right hemisphere regions were more extensive (12). To conclude, these MRI studies have identified changes in the brain that are related to long COVID; the results of these studies were well complemented by those showing hypometabolism and hypoperfusion. These investigations also represent an important tool for use by clinicians in the exploration of cognitive dysfunction in patients with long COVID.

### Immune and glial interaction in COVID

The role of immune system and glial interactions was investigated in patients both in clinical trials and in postmortem samples. Etter et al. (13) have defined Neuro-COVID as the prevalence of neurological symptoms after SARS-CoV2 infection. They performed a cross-sectional clinical study (NCT04472013) that included clinical and imaging data and corresponding multidimensional characterization of immune mediators in the CSF and plasma of patients belonging to different Neuro-COVID severity classes. Signs of severe Neuro-COVID were found to be blood-brain barrier impairment, microglia reactivity, and a polyclonal B-cell response. In addition, COVID-19 patients showed decreased regional brain volumes associating with specific CSF parameters; despite undergoing a plasma cytokine storm, COVID patients’ CSF profile is a non-inflammatory one (13). These observations are pointing towards immune-mediated mechanisms responsible for severe Neuro-COVID.

Yang and coworkers (14) have profiled 65,309 single-nucleus transcriptomes from 30 frontal cortex and choroid plexus postmortem samples in 14 control individuals and 8 patients with COVID-19. They did not detect molecular traces of SARS-CoV-2 in the brain; instead, they found changes in the barrier cells of the choroid plexus and peripheral T cells infiltrating the parenchyma. These authors also showed that the microglial and astrocyte subpopulations in COVID-19 patients had features that resembled those seen in human neurodegenerative disease. Synaptic signaling of upper-layer excitatory neurons which are linked to cognition was significantly affected in COVID-19. Across cell types, perturbations associated with COVID-19 overlapped with those found in chronic brain disorders (14). This occurrence of similarities in brain changes in COVID-19 patients and those in neurodegenerative disease is worrisome and requires further investigation.

Seminal data regarding microglial immune activation in the brain of COVID-19 infected individuals was provided by a team that used post-mortem highly multiplexed spatial analysis in brain sections from 25 COVID-19 patients with severe neurological symptoms (15); substantial immune activation, as well as astrocytosis, axonal damage, and blood-brain barrier leakage were observed in the CNS of COVID-19 patients. In addition, viral antigens were detected in ACE-2 receptor-positive cells, which were enriched in the vascular compartment. Microglial nodules and the perivascular compartment were seen to represent COVID-19-specific, microanatomic immune niches with context-specific cellular interactions enriched for activated CD8^+^ T cells. Activated T cells showing possible bias toward exhaustion were found in microglial nodules. Patients with COVID-19 had a larger number of amyloid precursor protein deposits, indicating axonal damage. This work highlights the vascular compartment as a key site of immune activation and viral detection in COVID-19 brains. It also provides a framework for understanding the neurological comorbidities of SARS-CoV-2-infection, emphasizes specific cellular brain immune clusters involved in a COVID-19-specific neuroinflammatory pattern, and can help identify targets for immune intervention (15). All these data indicate that neuroinflammatory changes such as blood–brain barrier (BBB) leakage, T-cells dysregulation, astrocytosis, microglial activation, and axonal damage may play a significant role in the brain dysfunction seen in patients with long COVID. In addition, they suggest an important role for endothelial cells.

### COVID-19, complement, and endothelial cells

**Figure 1:**
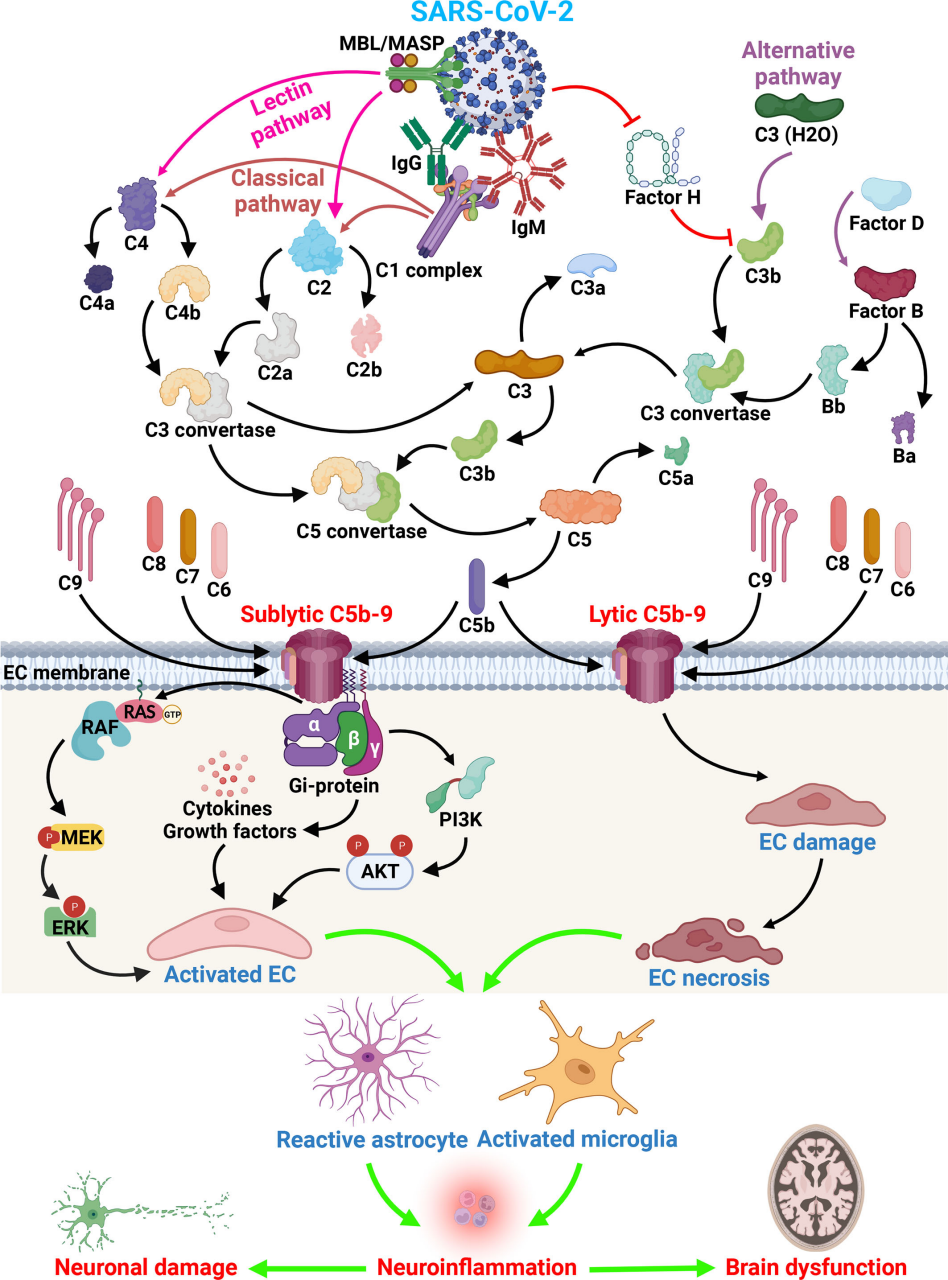
SARS-CoV-2 and complement activation effect on brain endothelial cells. Immunoglobulins raised after SARS-CoV-2 infection can have direct effects on cells, but also indirect effects as shown by the activation of the complement system. All three activation pathways (classical, alternative and lectin) can be activated by the virus and lead to the formation of C3 convertase and C5 convertase. The formation of the terminal complement complex C5b-9 leads to the EC lysis or activation. Insertion of sublytic C5b-9 on EC lead to the activation of cell signaling pathways important in cell proliferation (ERK1 and PI3K pathway), the release of growth factors (PDGF, bFGF, PIGF) and cytokines (IL-1, IL-6, IL-8, MCP1). Subsequent to EC activation and lysis the infiltration of macrophages and T cells occurs, followed by activation of astrocytes and microglia and leakage of plasma proteins as a consequence of BBB changes and subsequent neuronal dysfunction. All these changes lead to brain dysfunction seen in long COVID syndrome (Created with BioRender.com).

Kaneko et al. (16) have shown that COVID-19 can directly infect endothelial cells of the brain, potentially promoting clot formation and stroke. They have also shown that brain endothelial cells are susceptible to direct SARS-CoV-2 infection through flow-dependent expression of ACE-2. Viral S protein binding triggers a unique gene expression profile in the brain endothelium that may explain the association of SARS-CoV-2 infection with cerebrovascular events. Binding of SARS-CoV-2 S protein has been shown to trigger 83 unique genes in human brain endothelial cells, including upregulation of complement component C3 (16). The C3 factor plays a central role in the complement cascade, participating in both the alternative and classical pathways (17). C3 is cleaved into C3a and C3b, is followed by the binding of C3b to cellular surfaces and ultimately by C5b-9 assembly (**Figure 1**), which can lead to cell lysis or to sublytic C5b-9 assembly and target cell activation (17). C3-deficient mice show reduced neuroinflammation in response to stroke (18, 19). In human subjects, there is evidence for the usefulness of plasma exosomes enriched in C3b as a biomarker of cerebrovascular diseases. In fact, the findings of Elahi and coworkers have implicated both the classical and alternative complement pathways as well as C3b and the C5b-9 terminal complement complex (membrane attack complex [MAC]), in the inflammatory endothelial changes contributing to the white matter hyperintensities seen in older patients (20). The proinflammatory effects of sublytic C5b-9 on endothelial cells are also well documented (21) (**Figure 1**).

Wenzel et al. (22) have obtained evidence that in brains of SARS-CoV-2 patients, endothelial cells are infected, and that the main protease (M^pro^) of SARS-CoV-2 cleaves NEMO, the essential modulator of nuclear factor-κB. The M^pro^ are a class of highly conserved cysteine hydrolases in β-coronaviruses Through NEMO annihilation, M^pro^ was able to induce the death of human brain endothelial cells in mice. Deletion of receptor-interacting protein kinase (RIPK) 3, a mediator of regulated cell death, blocked the disruption of the blood–brain barrier caused by NEMO ablation. Importantly, a pharmacological inhibitor of RIPK signaling prevented the M^pro^-induced endothelial cell death, suggesting that RIPK could be a potential therapeutic target for treating this COVID-19 neuropathology (22).

Further progress on the role of endothelial cells in the neurological effects of SARS-CoV-2 has been made by Lee and coworkers (23, 24). These authors examined post-mortem tissue of patients who died during the COVID-19 pandemic in 2020. In their later study (24) they amplified their previous observations to further describe the new vascular and immunological changes associated with microvessels in COVID-19 brains. As compared to the control brains, the brain tissue of patients who died from COVID-19 showed a significant increase in the extravasation of fibrinogen, indicating widespread disruption of the BBB. In addition, the authors noted platelet accumulation, an increased expression of platelet endothelial cell adhesion molecule 1 (PECAM-1), and increased tissue factor and von Willebrand factor in the COVID-19 brains. To determine whether the endothelial cell damage was immune-mediated, they examined the brains post-mortem for the presence of immunoglobulins and complement components and found deposits of IgG, IgM, C1q, C4d, and C5b-9, which point to the activation of the classical complement pathway and the occurrence of immune-mediated injury to the endothelial cells. The endothelial cell antigen(s) against which this immune response was developed has not yet been identified. The authors speculate that the antibodies are directed against an antigen on the endothelial cells or against the immune complexes formed by the antibodies and spike protein that may bind to the ACE-2 receptors on the endothelial cells. The endothelium in the brain can be activated by cytokines such as interleukins or TNF-α, which induce cell adhesion molecules and leakage of the blood–brain barrier. An activated endothelium and blood–brain barrier disruption can then result in an infiltration of immune cells. Lee et al. have shown an infiltration of CD3- or CD8-positive T cells and CD68-positive macrophages in the perivascular spaces in COVID brains (24). They also found morphological signs of neuronophagia in the hindbrain, suggesting the occurrence of neuronal cell death and phagocytosis by microglia (24).

The resulting formation of the C5b-9/MAC could contribute to the endothelial cell (EC) lysis, either by direct or bystander cell lysis (17) (**Figure 1**). When the sublytic C5b-9 is deposited on its surface, the endothelium gains procoagulant and pro-adhesive properties as well as exhibiting the secretion of von Willebrand factor, expression of P-selectin (25), IL-1 α release, a secondary increase in tissue factor expression (26, 27), and the production of monocyte chemoattractant protein-1, IL-1β, and IL-8 (28). C5b-9 assembly on TNF-α-stimulated ECs results in enhanced neutrophil adhesion through the induction of E-selectin and ICAM-I expression (29). In addition, sublytic C5b-9 induces ERK1 activation and the release of basic fibroblastic growth factor and platelet-derived growth factor from ECs, thus promoting C5b-9-induced cellular proliferation (21, 30). Fosbrink and coworkers eloquently demonstrated that the activation of PI3K/Akt/FOXO1 pathway was required for C5b-9-induced cell cycle activation in EC (31) (**Figure 1**). FOXO1, a forkhead transcription factor, was phosphorylated at Ser-256 and inactivated after sublytic C5b-9 stimulation. Silencing FOXO1 expression with specific siRNA stimulated EC proliferation and regulated angiogenic factor release (31). These data suggest that sublytic C5b-9 regulation of the cell cycle activation in ECs through the Akt pathway is dependent on the inactivation of FOXO1. RGC-32 silencing in primary human ECs abrogated the ability of C5b-9 to induce the cell cycle and CDC2 activation, while suppressing the PI3K/Akt/FOXO1 pathway and altering the C5b-9-induced expression profile of various growth factors; as such, loss of RGC-32 in human ECs translated in diminished release of leptin, PIGF, and RANTES and increased release of IL-8, TIMP-1, and VEGF-D (32). By using an oligonucleotide expression array and ECs in which RGC-32 expression had been silenced, we have shown that the genes differentially regulated by RGC-32 are involved in actin cytoskeletal organization, cell adhesion, response to stress, and cell cycle regulation (25). Taken together, these data suggest that complement activation and both lytic and sublytic C5b-9 effects on endothelial cells contribute to the pathological vascular changes seen in the brains of patients with COVID-19.

To conclude, long COVID can have significant and devastating neurological effects on the brain of patients with COVID, even in those which do not require hospitalization. A significant number of research papers have shown and implicated multiple pathophysiological mechanisms. As discussed above, cognitive dysfunction is a major complaint in patients with long COVID and is mediated at least in part by elevated CCL11 levels by spike protein induced TLR4. In addition, endothelial cells and neuroinflammatory changes involving BBB, T-cells, astrocytosis, microglial activation, and axonal damage play a significant role in the brain dysfunction seen in patients with long COVID. The contribution of complement activation and both lytic and sublytic C5b-9 effects on endothelial cells to the pathological vascular changes seen in the brains of patients with COVID-19 is now considered very important and led to clinical trials using C5 blockade with monoclonal antibodies or drugs targeting C5, C5a, or C5aR1 are still ongoing (33). Preliminary results indicate that early timing of C5 blockade may be crucial. Targeting the cognitive and cerebrovascular effects of COVID-19 and the pathogenic events involved, including complement activation, can potentially lead to effective therapies for long COVID.

## Author contributions

SV, AT, VR and HR designed the study. SV, AT, JC, VR and HR wrote the manuscript. All authors approved the manuscript.
